# Influenza virus: 16 years’ experience of clinical epidemiologic patterns and associated infection factors in hospitalized children in Argentina

**DOI:** 10.1371/journal.pone.0195135

**Published:** 2018-03-29

**Authors:** Angela Gentile, Maria Florencia Lucion, Maria del Valle Juarez, Ana Clara Martinez, Viviana Romanin, Julia Bakir, Mariana Viegas, Alicia Mistchenko

**Affiliations:** 1 Department of Epidemiology, Ricardo Gutiérrez Children’s Hospital, Buenos Aires, Argentina; 2 Department of Virology, Ricardo Gutiérrez Children’s Hospital, Buenos Aires, Argentina; Defense Threat Reduction Agency, UNITED STATES

## Abstract

**Background:**

Influenza is an important cause of acute lower respiratory tract infection (aLRTI), hospitalization, and mortality in children. This study aimed to describe the clinical and epidemiologic patterns and infection factors associated with influenza, and compare case features of influenza A and B.

**Methods:**

In a prospective, cross-sectional study, patients admitted for aLRTI, between 2000 and 2015, were tested for respiratory syncytial virus, adenovirus, influenza, or parainfluenza, and confirmed by fluorescent antibody (FA) or real-time polymerase chain reaction (RT-PCR) assay of nasopharyngeal aspirates.

**Results:**

Of 14,044 patients, 37.7% (5290) had FA- or RT-PCR-confirmed samples that identified influenza in 2.8% (394/14,044; 91.4% [360] influenza A, 8.6% [34] influenza B) of cases. Influenza frequency followed a seasonal epidemic pattern (May–July, the lowest average temperature months). The median age of cases was 12 months (interquartile range: 6–21 months); 56.1% (221/394) of cases were male. Consolidated pneumonia was the most frequent clinical presentation (56.9%; 224/394). Roughly half (49.7%; 196/394) of all cases had previous respiratory admissions; 9.4% (37/394) were re-admissions; 61.5% (241/392) had comorbidities; 26.2% (102/389) had complications; 7.8% (30/384) had nosocomial infections. The average case fatality rate was 2.1% (8/389). Chronic neurologic disease was significantly higher in influenza B cases compared to influenza A cases (p = 0.030). The independent predictors for influenza were: age ≥6 months, odds ratio (OR): 1.88 (95% confidence interval [CI]: 1.44–2.45); p<0.001; presence of chronic neurologic disease, OR: 1.48 (95% CI: 1.01–2.17); p = 0.041; previous respiratory admissions, OR: 1.71 (95% CI: 1.36–2.14); p<0.001; re-admissions, OR: 1.71 (95% CI: 1.17–2.51); p = 0.006; clinical pneumonia, OR: 1.50 (95% CI: 1.21–1.87); p<0.001; immunodeficiency, OR: 1.87 (95% CI: 1.15–3.05); p = 0.011; cystic fibrosis, OR: 4.42 (95% CI: 1.29–15.14); p = 0.018.

**Conclusion:**

Influenza showed an epidemic seasonal pattern (May–July), with higher risk in children ≥6 months, or with pneumonia, previous respiratory admissions, or certain comorbidities.

## Introduction

Respiratory tract infections are one of the main causes of medical visits in pediatrics [1,2], constituting a major public health problem. Viruses are the most commonly implicated agents in acute lower respiratory infection (ALRI) in children <5 years old [3], the most important being respiratory syncytial virus (RSV), influenza A and B, adenovirus (AV), and parainfluenza 1, 2, and 3; however, other pathogens such as human metapneumovirus and rhinovirus have more recently been recognized as causes of ALRI [3]. Of these, influenza has distinctive characteristics regarding its epidemiologic, genetic, and antigenic properties, as well as its impact on the general population. Influenza remains an important infectious disease across the globe and causes considerable morbidity and mortality [1].

Influenza has an estimated annual attack rate of 5–10% in adults and 20–30% in children, with reports of severe illness in 3–5 million cases and almost half a million deaths globally [1]. It is estimated that, annually, 60% of children <1 year old and 50% of children <5 years old experience an acute respiratory infection of some type, including obstructive bronchitis and pneumonia [1,2]. Modeling studies suggest that children <2 years old have high rates of hospitalization that are attributable to influenza [1,4,5]. Though seasonal influenza can affect any population, children aged <2 years have the highest risk of complications [1]. Estimation of the true influenza burden remains a challenge. The data collected through influenza surveillance and case-finding represent only a fraction of those infected with influenza because not all of those infected with influenza will seek a medical diagnosis and be reported through their country’s influenza surveillance program [6]. A central feature of influenza is its antigenic variability, which is the result of two main mechanisms: antigenic drift and antigenic shift. The latter occurs only with the influenza A virus, giving rise to viral strains unknown to the human immune system and potentially resulting in pandemics [7].

At the Ricardo Gutiérrez Children’s Hospital in Buenos Aires, Argentina, there has been active epidemiologic surveillance of respiratory viruses in hospitalized patients with ALRI since 2000. This program allows observation of the distribution of viral agents in hospitalized patients, detection of outbreaks, and establishment of control measures, as well as a network of adequate containment. Active epidemiologic surveillance is an important tool to assess the impact of respiratory viruses in a pediatric population. Influenza mortality and morbidity in Latin America and the Caribbean is possibly underreported and thus information is not readily available. In Latin America and the Caribbean, most reported data come from a diagnosis of influenza-like illness and the true burden of influenza remains obscure because specific diagnostic tools to confirm etiology are often not used [8]. In Argentina, respiratory infections are among the five leading causes of death in children <5 years old [2]. The aims of this study were to describe the clinical and epidemiologic pattern of influenza and factors associated with influenza and to compare the characteristics of influenza A and B cases.

## Materials and methods

A prospective, epidemiologic, cross-sectional study was conducted in accordance with the World Medical Association Declaration of Helsinki International Code of Ethics for experiments involving humans; the privacy rights of patients were always observed. Patient’s informed consent is not applicable in this study because the data were obtained from a routine epidemiological surveillance activity included in the framework of Argentinian Law 15465/60. The study was approved by the Ethics and Research Committees of the Ricardo Gutiérrez Children’s Hospital. This study will not affect human rights, nor will it cause damage to the environment, animals and/or future generations. All patients admitted for ALRI acquired in the community were detected through the active Epidemiological Surveillance Program at the Ricardo Gutiérrez Children’s Hospital, Buenos Aires, between 2000 and 2015. The study included all children (≤18 years of age) who were hospitalized for ALRI with bronchiolitis or pneumonia. The definition of pneumonia in this study was acute infection of the pulmonary parenchyma with clinical signs of alveolar occupation. All admitted children with ALRI underwent viral testing for diagnosis. Data were collected with a specific case-reporting form, including date of admission, demographics (e.g., age, sex, the patient’s city of residence), clinical presentation (bronchiolitis, pneumonia), previous hospitalizations related to respiratory disease, readmission for the same episode, comorbidities, history of close contact with any acute respiratory disease of probable viral cause (runny nose, cough, and/or fever), neonatal respiratory pathology, complications during hospitalization and patient’s course in hospital (discharge, transfer to another center, death), treatment, and length of stay. Comorbidities noted included chronic or recurrent respiratory disease, malnourishment, congenital heart disease, genetic or neurologic diseases, and immunodeficiency.

Regarding chronic or recurrent respiratory disease, the presence of any of the following conditions was recorded: recurrent obstructive bronchitis or asthma, gastroesophageal reflux disease, cystic fibrosis, bronchopulmonary dysplasia, recurrent laryngitis, and pneumonia; recurrent obstructive bronchitis was defined as the occurrence of two or more broncho-obstructive episodes. As complications, nosocomial infections, sepsis, persistent atelectasis, pneumothorax, pleural effusion, bullae, abscesses, ear infections, diarrhea, convulsions, and meningitis were considered. Nosocomial infection was defined as an exacerbation of respiratory symptoms not present at admission (even if incubating), presenting in patients hospitalized for ≥48 hours, expressed as fever, increased oxygen requirement, or changes to radiologic pattern.

### Diagnostic methods

The clinical and radiologic diagnosis of bronchiolitis and pneumonia was conducted in accordance with the guidelines of the Argentinian Society of Pediatrics [9]. Virologic diagnosis was performed by indirect fluorescent antibody of nasopharyngeal aspirates in all cases. Real-time polymerase chain reaction (RT-PCR) assay became available from 2009 and was used in addition to the indirect fluorescent antibody method for detection of RSV, AV, influenza A and B, and parainfluenza virus. For all diagnoses, commercially available methods were used.

### Statistical analysis

Epi Info™ version 7 (US Centers of Disease Control and Prevention, Atlanta, GA, USA) was used for data analysis. The categorical variables were analyzed using the χ2 test with Yates correction. We used the Wilcoxon test for comparing median age. The measure of association used was the odds ratio (OR) with a confidence interval (CI) of 95%. A bivariate analysis was performed first to identify the significant association. Following this, a multivariate analysis was carried out to establish independent predictors using logistic regression model of SPSS software version 15.0 (SPSS Inc., Chicago, IL, USA). The following variables were included in the model: age ≥6 months, chronic neurologic disease, previous admissions for respiratory causes, readmissions, presentation with clinical pneumonia, immunodeficiency, cystic fibrosis, congenital heart disease, and previous admissions for respiratory causes. A probability of less than 0.05 was considered significant.

## Results

**Fig 1:**
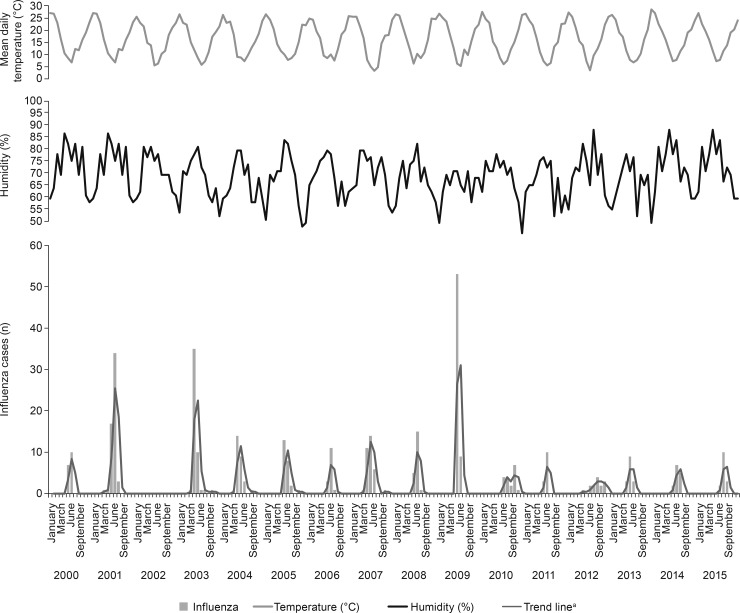
Seasonal distribution of confirmed influenza cases in hospitalized children in Buenos Aires, Argentina, 2000–2015 (n = 394). ^a^Moving average trend line, period = 2.

A total of 14,044 patients were included, 37.7% (5290) of whom had positive samples; influenza represented 2.8% (394/14,044); of these, 91.4% (360/394) had influenza A and 8.6% (34/394) had influenza B. The influenza activity data (Fig 1) showed a seasonal epidemic pattern from May to July (interquartile range [IQR] of epidemiologic weeks [EW] of viral activity: 24–29), with peaks of major activity matching low temperatures and higher humidity. Influenza incidence remained high throughout the winter until spring. The peaks were observed between EW 25–26 (June), except during 2003, which showed earlier peaks from EW 21 (May). No influenza cases were recorded in 2002.

**Fig 2:**
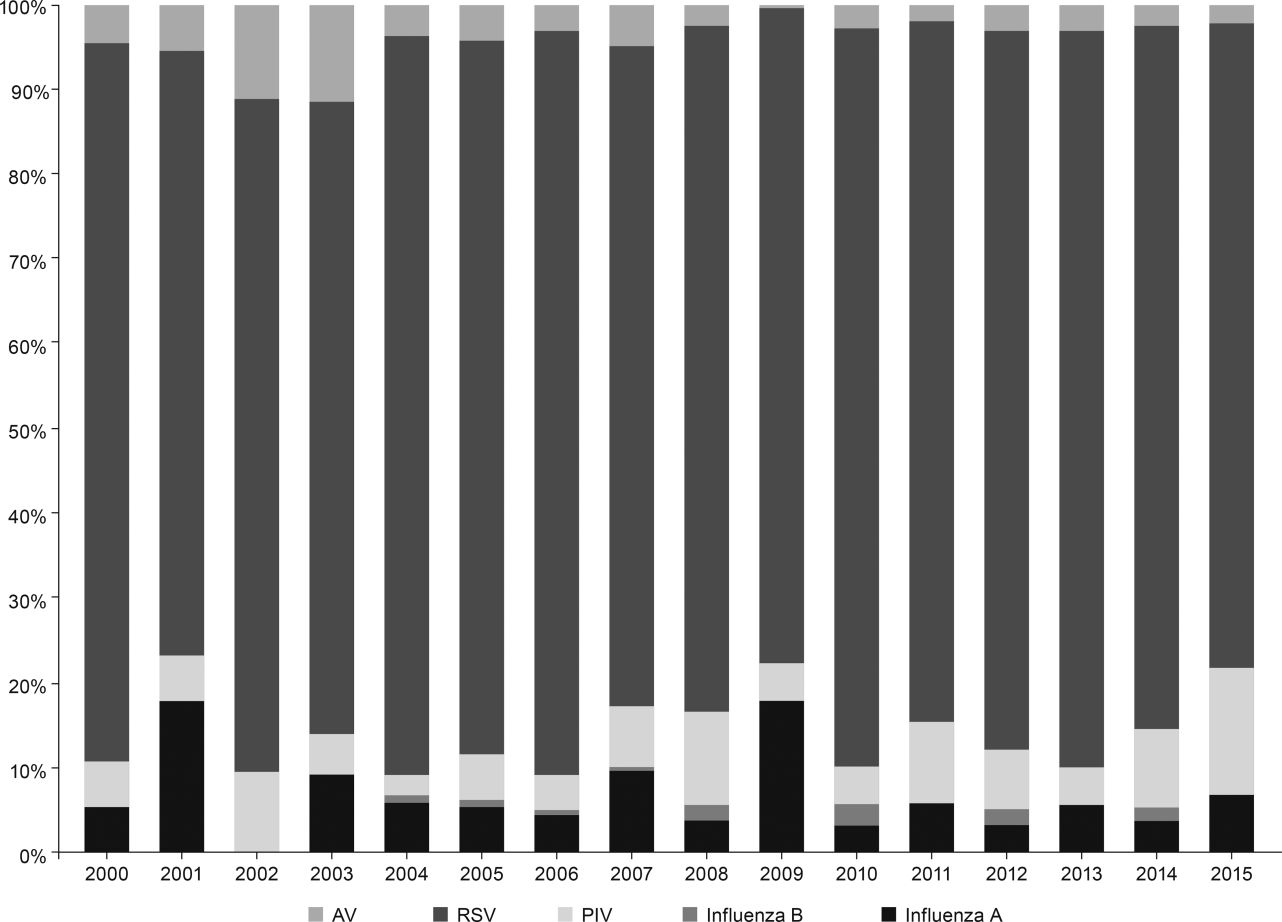
Annual distribution of confirmed viral pathogens in nasopharyngeal aspirates of hospitalized children in Buenos Aires, Argentina, 2001–2015 (n = 5290). AV, adenovirus; PIV, parainfluenza virus; RSV, respiratory syncytial virus.

The annual distribution of respiratory viruses varied somewhat over the past 16 years; however, RSV was consistently the most frequently reported pathogen (Fig 2). The median age of influenza cases was 12 months (IQR: 6–21 months) and 56.1% were male (Table 1). Consolidated pneumonia was the most frequent clinical presentation (56.9%). Comorbidities were found in 61.5% (241/392) of patients. Complications were detected in 26.2% (102/389) of patients. Each patient may have had >1 comorbidity or complication. Comparing influenza A and B, chronic neurologic disease was significantly higher in influenza B cases (p = 0.030) (Table 1).

**Table 1:** Clinical and demographic characteristics of hospitalized children with confirmed influenza A and B in Buenos Aires, Argentina, 2000–2015 (n = 394).

Confirmed influenza (A: n = 360; B, n = 34)	Influenza A n (%)	Influenza B n (%)	OR (95% CI)	p value
Sex (male)	200 (55.6)	21 (61.7)	0.77 (0.37–1.59)	0.485
**Median age: 1 year (IQR: 6–21 months)**	12 (6–21)	17.5 (8–35)		0.082
<3 months (vs ≥3 months)	34 (9.4)	0 (0)	Undefined	–
<6 months (vs ≥6 months)	80 (22.2)	5 (14.7)	1.65 (0.62–4.42)	0.309
<12 months (vs ≥12 months)	174 (48.3)	13 (38.2)	1.51 (0.73–3.11)	0.260
<24 months (vs ≥24 months)	278 (77.2)	24 (70.6)	1.41 (0.64–3.07)	0.382
>24 months to ≤5 years (vs >5 years)	53 (14.7)	6 (17.6)	0.80 (0.31–2.03)	0.648
Prematurity (A:358; B:34)	71 (19.8)	3 (8.8)	2.55 (0.75–8.60)	0.117
Neonatal respiratory pathology (A:358; B:34)	67 (18.7)	5 (14.7)	1.33 (0.49–3.57)	0.564
Immunodeficiency (A:357; B:34)	18 (5.0)	4 (11.8)	0.39 (0.12–1.25)	0.104
Malnourished (A:357; B:33)	30 (8.4)	6 (18.2)	0.41 (0.15–1.08)	0.063
Previous admissions for respiratory causes (A:357; B:34)	177 (49.6)	19 (55.9)	0.77 (0.38–1.57)	0.483
**Comorbidities**^a^ **A: n = 219 / B: n = 22**				
Recurrent obstructive bronchitis	159 (72.6)	15 (68.2)	1.24 (0.48–3.18)	0.659
Chronic neurologic disease	31 (14.2)	7 (31.8)	0.35 (0.13–0.93)	0.030
Congenital heart disease	20 (9.1)	3 (13.6)	0.63 (0.17–2.34)	0.493
**Complications**^b^ **A: n = 91 / B: n = 11**				
Respiratory distress	31 (34.1)	5 (45.4)	0.62 (0.17–2.19)	0.457
Acute otitis media	23 (25.3)	2 (18.2)	1.52 (0.30–7.56)	0.607
Atelectasis	11 (12.1)	2 (18.2)	0.62 (0.12–3.24)	0.568
Sepsis	14 (15.4)	2 (18.2)	0.82 (0.16–4.19)	0.810

A, influenza A; B, influenza B; CI, confidence interval; IQR, interquartile range; OR, odds ratio.

^a^ Patients may have had >1 comorbidity.

^b^ Complication percentage calculations are based on n = 102 patients with complications.

While comparing influenza cases (influenza A and B) against the non-influenza ALRI cases (RSV, parainfluenza virus, AV), the following were independent predictors for influenza infection: age ≥6 months (compared with <6 months), OR: 1.88 (95% CI: 1.44–2.45); p<0.001 (Table 2); presence of chronic neurologic disease, OR: 1.48 (95% CI: 1.01–2.17); p = 0.041; previous admissions for respiratory causes, OR: 1.71 (95% CI: 1.36–2.14); p<0.001; readmissions, OR: 1.71 (95% CI: 1.17–2.51); p = 0.006; presentation with clinical pneumonia, OR: 1.50 (95% CI: 1.21–1.87); p<0.001; immunodeficiency, OR: 1.87 (95% CI: 1.15–3.05); p = 0.011; cystic fibrosis, OR: 4.42 (95% CI: 1.29–15.14); p = 0.018.

**Table 2:** Multivariate analysis of the probability of influenza diagnosis among all confirmed viral pathogens in nasopharyngeal aspirates of hospitalized children in Buenos Aires, Argentina, 2001–2015 (n = 5290).

Independent predictors	Influenza (n = 394)	Other pathogen (n = 4896)	Adjusted OR	95% CI	p value
Age ≥6 months	78.4% (309)	58.7% (2875)	1.88	1.44–2.45	<0.001
Chronic neurologic disease	9.6% (38)	4.4% (218)	1.48	1.01–2.17	0.04
Previous admissions for respiratory causes	49.7% (196)	29.5% (1444)	1.71	1.36–2.14	<0.001
Re-admission for the same episode	9.4% (37)	4.3% (213)	1.71	1.17–2.51	0.006
Pneumonia clinical presentation	56.9% (224)	40.2% (1968)	1.50	1.21–1.87	<0.001
Immunodeficiency	5.6% (22)	2.1% (103)	1.87	1.15–3.05	0.011

CI, confidence interval; OR, odds ratio.

## Discussion

The present study shows that, in Argentina, influenza incidence in hospitalized children was higher from winter through spring. Peaks of incidence in June correspond to periods of low temperature and high humidity, consistent with the seasonal influenza epidemiologic pattern in temperate regions [10]. However, earlier studies have associated peak influenza incidence with low temperature and low indoor humidity in temperate regions [11], and with relatively higher temperatures and higher humidity in tropical regions [11–14]. Thus, further studies are needed to understand the association of influenza epidemic patterns with low temperature and high humidity. We show that, although the annual distribution of respiratory viruses has varied over time, RSV has remained the most frequently reported pathogen. This is in line with our previous publication on clinical and epidemiologic patterns of RSV in pediatric populations [15]. The high peak of influenza in 2009 reflects the occurrence of the swine flu pandemic.

The present study found that 49.7% of patients with influenza reported comorbidities and 26.2% of patients had medical complications. Consolidated pneumonia was a predominant clinical characteristic among patients with confirmed influenza, and this has recently been documented elsewhere [16]. Patients with previous hospitalization for respiratory causes were more prone to be affected by seasonal influenza. No significant association was found when comparing cases of influenza A and B infection. Studies on pediatric and adult populations have compared demographic and clinical features of patients with influenza A versus influenza B, and have generally found few differences [17–19]. This study is based on an active epidemiologic surveillance carried out since 2000 in the Ricardo Gutiérrez Children’s Hospital, which is a tertiary center, and a national and international reference pediatric hospital serving the metropolitan area of Buenos Aires, Argentina. The value of the extensive experience gained by the same surveillance team using the same criteria over 16 years is demonstrated in the strength of our data. On the other hand, the Virology Laboratory is a national center of reference for diagnosis. To the best of our knowledge, this is the largest report of data (more than 14,000 specimens) from children hospitalized with ALRI in Argentina, documenting the clinical and epidemiologic pattern of seasonal influenza between 2000 and 2015. Marcone et al. have recently reported similar outcomes on the incidence of viral respiratory infection in a prospective cohort study including inpatient and outpatient Argentinian children ≤5 years old [20]. However, that study reported the respiratory viral incidence of only ~1800 patients between 2008 and 2010. Therefore, the current study has provided a broader estimation of influenza burden and seasonal influenza epidemiologic patterns in Argentinian children hospitalized with ALRI.

This study has the limitation of being a hospital-based study, and it is not possible to infer results for the general population. We also did not collect data of other sociodemographic variables that might influence the results, such as socioeconomic status, housing conditions, overcrowding, attendance at preschool or kindergarten, or number and age of cohabitants.

## Conclusion

Influenza in hospitalized children in Argentina showed a seasonal epidemic pattern (May–July) and mostly affected children ≥6 months old, as well as those who had pneumonia, previous admissions for respiratory causes, and certain comorbidities.
